# Cultivar, Trait and Management System Selection to Improve Soft-Red Winter Wheat Productivity in the Eastern United States

**DOI:** 10.3389/fpls.2020.00335

**Published:** 2020-03-31

**Authors:** Blake Russell, Carlos Guzman, Mohsen Mohammadi

**Affiliations:** ^1^Department of Agronomy, Purdue University, West Lafayette, IN, United States; ^2^Departamento de Genética, Escuela Técnica Superior de Ingeniería Agronómica y de Montes, Edificio Gregor Mendel, Campus de Rabanales, Universidad de Córdoba, Córdoba, Spain

**Keywords:** soft-red winter wheat, nitrogen use efficiency, yield components, grain number, kernel weight, nitrogen harvest index, glutenin subunits

## Abstract

Wheat growing regions and seasons are diverse, mandating different varietal adaptation and management practices. Grain yield is the primary target for soft-red winter (SRW) wheat, due to lower protein content requirements. The growing season for SRW wheat in the eastern United States takes up to 9 months under variable environments, highlighting the importance of variety and management. In this study, we present the results of a 2-year field-based investigation of yield response of 30 wheat lines to different nitrogen treatments by dissecting yield to its components. For 5 out of the 30 lines, we performed in-tissue nitrogen analysis. Spring nitrogen (N) treatments were two levels of 0 kg N ha^–1^ (low N) and 112 kg N ha^–1^ (high N). On average, application of 112 kg N in the spring, in addition to fall N fertilizer, increased phytomass by 22% at maturity, enhanced fertile tiller numbers by 16%, and increased grain yield by 18% that coincided with a 26% increase in grain number per unit area. N in the grains, or the nitrogen harvest index, was lower (36% of total) in high N than in low N (40% of total) treatment, which indicated plants did not increase the in-grain utilization of N. The 18% higher grain yield with 112 kg N treatment occurred without considerable change in grain N content. However, lines with greater biomass produced greater yields in low N. Therefore, increasing tiller numbers and grain numbers for SRW wheat are the targeted traits for improving grain yield under N management, with less emphasis on the utilization of N in grains because N content is not critically influential for the marketability of soft wheat grains.

## Introduction

Wheat cultivation occupies 22% of the major croplands globally, and covers the temperate latitude of both hemispheres, consisting of the Great Plains in United States, Canadian Prairie Provinces, western Europe, the Indus and the upper Ganges valleys, southern South America, eastern Africa, eastern China, southern Australia, and along the Kazakhstan and Russia border (Leff et al., 2004). Wheat grown throughout the world consists of either spring or winter wheat. Winter wheat requires a vernalization period to transition from vegetative to reproductive stage (Dubcovsky et al., 2006). The vernalization requirement is genotype specific, with variations in time (15–45 days) and temperature (0–5°C) (Crofts, 1989). Some wheat producing regions manage autumn-grown wheat that are not considered winter types. These regions use the mild but elevated winter temperatures to grow wheat for higher yield potential. Examples of these locations are Mexico, California, and parts of the Middle East. Winter wheat is typically not viewed as a cover crop but has dual grain and grazing purposes in targeted regions such as Oklahoma and Texas (Maulana et al., 2019).

A key characteristic of wheat is the unique properties of forming dough from flour (Shewry, 2009). Quality is indicated by the performance of a cultivar at specific protein levels for defined end use products (Bushuk et al., 1997) and viscoelastic properties (Shewry, 2009). Wheat classes are defined by grain hardness, protein content, and growth habit. Hard wheat has hard endosperm texture and higher protein content. Soft wheat has soft endosperm texture, low levels of damaged starch granule upon milling, and weaker dough strength that is suitable to make biscuits, cookies, and cakes (Bushuk et al., 1997). Protein composition in the endosperm is made of monomeric gliadins and polymer glutenins subunits (Porceddu et al., 1997). Glutenins are further divided into high molecular weight (HMW) and low molecular weight (LMW) subunits. The composition of high and low molecular weight glutenin subunits is the key quality determinant for dough (Bushuk et al., 1997). In addition to genetics, protein quantity and quality is dependent on environmental conditions (Luo et al., 2000; Cooper et al., 2001).

Management practices in wheat have substantial impacts on crop productivity and environmental stewardship. In both winter and spring wheat cropping systems, nitrogen (N) fertilizer applications are routinely applied pre-planting or during leaf formation (Zadoks 15) with additional N top-dress application in the stem elongation stage (Zadoks 30) or post-anthesis (Zadoks 69) (Woodard and Bly, 1998; Otteson et al., 2007). Developing a site-specific understanding for fertilizer expenses, environmental impacts such as leaching and volatilization, and efficient use of N by crops are pillars of crop profitability in relation to N management. Previous work by Koch et al. (2004) described the economic benefits for site-specific and environment-specific management practices for variable rate nitrogen applications, but further research is needed in the area of targeted genotype by environment by management practices for improved economic and environmental outcomes.

Nitrogen is necessary for growth of canopy, intercepting solar radiation, and photosynthesis in green tissues (Barraclough et al., 2014). Nitrogen use efficiency (NUE) is the amount of grain produced per unit of N available in the soil (Moll et al., 1982). In other words, the ability to increase grain yield per N applied. The two main components of NUE are uptake efficiency and utilization efficiency. Nitrogen uptake efficiency (NUpE) is the plant’s ability to absorb N available in the soil, and nitrogen utilization efficiency (NUtE) is the efficiency of which the absorbed N is utilized to produce grain (Moll et al., 1982). NUtE is also described as the ratio between crop yield and total N absorbed by the plant (Todeschini et al., 2016), indicative of the output of grain yield based on the amount of N taken up by the plant.

It is nearly impossible to identify and recommend a single variety that is the “best” across multiple environments due to the infinite interactions that can cause unstable phenotypic characteristics (Allard and Bradshaw, 1964). Yield is the most economically important trait, making both pre-planting and in-season crop management (Kirkegaard and Hunt, 2010) critical to maximize this market for growers and suppliers. The end-use quality traits such as protein content and endosperm texture are also influenced by N availability during plant growth. Farm profitability is primarily dependent on grain yield and quality. With approximately 7.8 million metric tons of soft-red winter wheat produced in the United States in 2018, accounting for ∼15% of total wheat production, it is paramount to strategically manage the cost and benefits to increase yields. The goal of our study was to identify traits responsive to N in a typical soft-red winter wheat breeding population under two contrasting N management and identify potential useful genetic solutions for the long term goal of managing wheat with reduced nitrogen fertilizer. To accomplish our goal, we evaluated grain yield, yield determining traits and N components under low N and high N environments and assessed protein quality.

## Materials and Methods

### Field Experiments and Nitrogen Management

Thirty experimental breeding lines, designated as PU01–PU30, from Purdue University’s soft-red winter wheat breeding program were selected based on their variation in grain yield (from 3,500 to 6,500 kg ha^–1^). These 30 lines were planted in the Purdue Agronomy Farm (40.43° N, 86.99°W) for two seasons: 2016–2017 and 2017–2018. The experimental layout included two N rates arranged in a split plot design with 4 blocks, where N rate was main-plot and line was sub-plot. Each experimental unit measured 1.22 m × 3.05 m, with 7 rows spaced 15 cm apart with a targeted planting density of 370 seeds m^–2^. The soil type at the Agronomy Research Farm is a combination of Rockfield silt loam (fine-silty, mixed, superactive, mesic Oxyaquic Hapludalfs), Fincastle silt loam (fine-silty, mixed, superactive, mesic Aeric Epiaqualfs), and Toronto silt loam (fine-silty, mixed, superactive, mesic Udollic Epaqulafs) (USDA Web Soil Survey). Experiments were planted in late September following corn and harvested late June of the following year. The experiments were planted using a Hege (Wintersteiger, Austria) drill planter and plots harvested with a Wintersteiger (Wintersteiger, Austria) plot harvester at physiological maturity.

In the fall, 224 kg ha^–1^ of mono-ammonium phosphate (11-52-0) was applied based on soil test (Mehlich-3) recommendations. The plot area was then chisel cultivated. Approximately 100 kg ha^–1^ of potassium chloride was added to the entire experimental area as recommended by soil analysis. Emergence began approximately 6 days after planting. Spring nitrogen applications of 112 kg N ha^–1^ of urea (46-0-0) was broadcast applied to the main plots, designed as high-N treatment, at stem elongation (Zadoks 30) growth stage. Prior to application, urea was treated with Limus (BASF, Germany), a urease inhibitor which prevents urea from being broken down via urease enzymes and lost through volatilization. The main plots, designated for low-N treatment, received zero spring N. Herbicide (Harmony Extra [thifensulfuron + tribenuron], DuPont, 35 g ha^–1^) was applied in mid-April to minimize weed pressure. Weather information including average monthly precipitation and temperature, as per iClimate (2019), are shown in Supplementary Table S1.

### Agronomic Traits

Days to heading (HD) and days to physiological maturity (MD) were recorded when 50% of the plot showed head emergence and maturity, respectively, and expressed as the number of days from January 1 of the current year. Plant height (PLH), from the ground to the top of the uppermost spikelet, was measured at four locations within the plot at physiological maturity. Thousand kernel weight was measured and the average weight for a single kernel was calculated (KW). Grain yield (YLD) was measured on a whole plot basis, corrected for 13% moisture.

The aboveground biomass (BIO) was estimated by cutting 0.25 m × 0.30 m (2 rows) from the middle of each plot for all treatments at heading (Zadoks 58), anthesis (Zadoks 60–68), and maturity (Zadoks 91) and dried to constant weight. Number of spikes per cut area (NS) was estimated by averaging the count of spikes at heading, anthesis, and maturity from the samples of cut area (0.25 m × 0.30 m). Yield component traits were measured from the same cut area sample at physiological maturity. Five random spikes were chosen to measure spike length (SPL), and hand-threshed to obtain the number of kernels per spike (KNS), kernel weight per spike (KWS), and grain number per cut area (GN). Fruiting efficiency (FE) was calculated by the number of kernels produced by each spike divided by the spike weight at anthesis. Lastly, harvest index (HI) was determined by the dividing the grain yield by the aboveground biomass at maturity.

We chose 5 out of 30 lines, based on earlier yield data, to analyze N concentration in biomass and grain. These lines showed a range of grain yield over 5 years and three locations in Indiana. The entire aboveground biomass (phytomass) was analyzed at heading and anthesis. At maturity once leaf senescence was complete, plant biomass was divided into grain and leaves plus straw. All samples were dried for 72 h at 49°C.

Plant samples were ground with cutting mill (Model E3703, Eberbach Corp., Bellevile, MI, United States) and UDY grinder (Udy Corp., Fort Collins, CO, United States) and passed through a 1.0 mm screen. Thirty milligrams of each sample were sent for flash combustion analysis (Flash EA 112 Series, CE Elantech, Lakewood, NJ, United States). The N concentration of phytomass at heading (NCPH) and anthesis (NCPA) were measured on whole plant samples. The nitrogen concentration of phytomass at maturity (NCPM) was measured on leaf and straw tissues. The nitrogen concentration of grains at maturity (NCGM) was measured on the grain samples.

For NUE measurement, we adopted the methods presented by Moll et al. (1982), and Foulkes et al. (2009).

$$\mathrm{NUE}=\frac{\text{Grain}\,\text{dry}\,\text{matter}}{\text{Available}\,\text{N}}$$

where Grain dry matter is the grain yield (kg ha^–1^) of plots at maturity (Zadoks 92), and available N, based on the formula, is the nitrogen available from the soil and fertilizer. Residual N was not tested and is not included in the study and calculation of NUE. In this estimation, instead of available N, we used the amount of N applications in each treatment. Both low-N and high-N environments received the same fall N application of 25 kg N ha^–1^ as monoammonium phosphate. A spring N application of 112 kg ha^–1^ N was applied to the high-N environment only. The total N supplied in low-N environment was 25 kg ha^–1^ N, while the total N supplied in the high-N environment was 137 kg ha^–1^ N. N uptake was calculated as the total nitrogen in the aboveground biomass including grain. NUtE was measured as grain dry matter produced per gram of plant N uptake. Nitrogen harvest index (NHI) was estimated as amount of nitrogen that was recovered in grains relative to overall N uptake of the plants.

### Phenotyping Grain and Flour Characterization

A subsample of grains from each N environment were subjected to Single Kernel Characterization System 4100 (SKCS) (Perten Instruments, Sweden) analysis. A single replicate was performed for each linein each N environment. The SKCS weighs and crushes individual kernels and converts the force-crush profile to a unit-less Grain Hardness Index (GHI). Whole-meal flour samples were also prepared with a UDY Cyclone mill (Udy Corp., Fort Collin, CO, United States) with a 0.5 mm screen. Sodium dodecyl sulfate (SDS) sedimentation volume was carried out according to the modified protocol described in Pena et al. (1990) using 1 g of flour.

### Glutenin Subunits and the Rye Translocation

Allelic variation of glutenin subunits and the presence or absence of the rye translocation were evaluated by sodium dodecyl sulfate polyacrylamide gel electrophoresis (SDS-PAGE) for all 30 lines following method described by Peña et al. (2004).

### Statistical Analysis

Combined year analysis of variance (ANOVA) was performed with PROC GLM in SAS 9.4 (SAS Institute, Cary, NC, United States) similar to the model presented by Iannucci et al. (2008), where sources of variations are year, nitrogen, year × nitrogen interaction, genotype, year × genotypes, nitrogen × genotypes, and year × nitrogen × genotype interaction effects, each tested against appropriate error term (Table 1).

**TABLE 1:** ANOVA for year (Y), nitrogen level (N), and genotype (G).

		Grain yield (YLD)		
Source of variation	d.f.	Mean square (× 10^4^)	*F*-value	Pr > *F*
Year (Y)	1	1286	5.23	ns
Residual 1	6	246		
N levels (N)	1	11144*	12.75*	*
Y × N	1	40.4	0.05	ns
Residual 2	6	874		
Genotype (G)	29	281***	8.31***	***
Y × G	29	132***	3.89***	***
N × G	29	53.5*	1.58*	*
Y × N × G	29	43.7	1.29	ns
Residual	348	33.9		
Total	479			

Significance: <0.001 = ***, ≤0.05*, and >0.05 = ns.

(1) $$\begin{array}{rcl} Y{}_{ijkl} & = & \mu +\mathrm{Yr}{}_{i}+\mathrm{rep(Yr)}{}_{li}+N{}_{j}+\mathrm{NYr}{}_{ji}+\mathrm{rep}{}^{*}\mathrm{N(Yr)}{}_{lji}\\ & & +G{}_{k}+\mathrm{GYr}{}_{ki}+\mathrm{GN}{}_{kj}+\mathrm{GNYr}{}_{kji}+\varepsilon {}_{ijkl} \end{array}$$

Where *Y*_ijkl_ is the phenotypic observation of the *l*^th^ replicate of the *k*^th^ genotype, in the *j*^t^*^h^* nitrogen treatment, observed in the *i*^th^ year. μ is the grand mean, *Yr*_i_ is the effect of *i*^th^ year, *rep(Yr)*_li_ is the effect of the *l*^th^ replicate in the *i*^th^ year. The effect of year was tested against *rep(Yr)*_li_. *N*_j_ is the effect of the *j*^th^ nitrogen treatment and *NY*_ji_ is the interaction effect of the *j*^th^ nitrogen level with the *i*^th^ year. These two terms were tested against the interaction effect of nitrogen by replicate within the year [*rep^∗^N(Yr)*_lji_]. *G*_k_ represents the effect of the *k*^th^ genotype. Remaining interactions were tested against the residual error. Tukey’s studentized range test (HSD) was implemented for comparison of means using the MEANS statement in PROC GLM (SAS 9.4) and significant differences reported with *p* < 0.05.

Least squares means was estimated using ‘*lsmeans*’ package (Lenth, 2016) in R environment (R Core Team, 2019) for genotypes and N levels with combining years and implemented for phenotypic analysis. Heritability, in the broad sense (*H*^2^) (Nyquist, 1991; Piepho and Möhring, 2007), was estimated for each nitrogen environment by restricted maximum likelihood (REML) variance and covariance components using PROC MIXED (SAS Institute Inc., 2013) with random effect model in equation 2.

(2) $$H^{2}=\frac{\sigma_{\text{g}}^{2}}{\sigma_{\text{g}}^{2}++\frac{\sigma_{\text{gy}}^{2}}{y}+\frac{\sigma_{\varepsilon }^{2}}{y\,r}}$$

With $\sigma_{\text{g}}^{2}$ representing variance component of genotype (genetic variance), $\sigma_{\text{gy}}^{2}$ the variance component of genotype × year interaction, and finally $\sigma_{\varepsilon }^{2}$ the residual error. Denominators represent years (*y* = 2), and replications (*r* = *4*). Pearson’s correlations were calculated for low-N and high-N environments separately using *cor* function in R environment (R Core Team, 2019). The linear relationship among measured traits was evaluated by Pearson’s correlation coefficient (*r*). Principal component biplot analysis was used to visualize relationships among traits and lines by using the *‘factoextra’* (Kassambara and Mundt, 2016) package and *‘factoMineR’* (Lê et al., 2008) package in R environment (R Core Team, 2019).

## Results

### Agronomic Traits

On average, the lines took approximately 130 days (from first of January) to head, and 168 days to reach physiological maturity (Supplementary Table S2). N effect was significant on biomass accumulated at physiological maturity (Supplementary Table S3). For example, biomass at maturity (BIO_MD_) was ∼22% greater in high N compared with low N.

The effects of G and N × G were significant for number of spikes (NS) (Supplementary Table S3). We observed correlations of *r* ≥ 0.21 between NS and BIO_MD_ in both N treatments (Supplementary Table S4), as more tillers produces more biomass. The lines showed variations in their number of tillers and biomass (Supplementary Table S2). PU10 and PU14 showed an average of approximately 60 NS across both N treatments, and BIO_MD_ greater than 95 g (Supplementary File S1). In comparison, PU21 and PU29 averaged 43 NS and BIO_MD_ of 87 and 88 g, respectively, showing a difference of 20 spikes and 10 g of biomass per cut area.

Number of spikes had the highest significant positive correlation observed with yield (*r* = 0.64^∗^ in low N; *r* = 0.36^∗^ in high N). On average, 8 more effective spikes per sampled area were observed in high N compared to low N, which resulted in 275 more kernels per sampled area in high N compared to low N (Supplementary Table S2). The grain number per unit area was a result of NS and effective tillers, which in our study, was significantly impacted by N. However, the weight of individual kernels was unaffected by N treatment (Supplementary Table S3). The mean KW was 36 mg, with a range of 25–47 mg across lines and environments (Supplementary Table S2). PU14 was the only line to have a KW above 40 mg in low N and high N (Supplementary File S1). We observed a negative correlation between GN and KW under both treatments (*r* = −0.34 low-N; *r* = −0.30 high-N) (Supplementary Table S4).

The effect of N, G, Y × G, and N × G were significant on YLD (Table 1) and the interaction of Y × N was not significant. On average, YLD was 976 kg ha^–1^ less in low N compared to high N (Supplementary Table S2). In the high-N treatment, YLD had a mean of 6,335 kg ha^–1^ and ranged between 3,799 and 8,090 kg ha^–1^. Difference in YLD resulted from producing more GN per treatment based on NS where N, G, N × G, and Y × N × G had significant effects on GN (Supplementary Table S3). Y, G, and G × Y had significant effects on HI. Across genotypes in environments, HI ranged from 0.21 to 0.55 (Supplementary Table S2). The 5 lines selected for in-tissue N analysis revealed a range of grain yield. For example, PU08, PU10, and PU15 exhibited YLD greater than the mean across both environments, and PU17 and PU21 exhibiting less YLD than average (Table 2).

**TABLE 2:** Nitrogen analysis and grain quality assessment of five subset lines.

	PU08	PU10	PU15	PU17	PU21
***Low-N***					
Yield (kg ha^–1^)	5,698	5,527	5,874	4,696	4,928
NUE (kg ha^–1^ grain/kg ha^–1^ N supply)	227.96	221.10	235.01	187.84	197.12
NCPH (mg g^–1^)	10.5	11.0	11.4	12.4	9.9
NCPA (mg g^–1^)	8.5	8.4	9.0	8.8	8.5
NCPM (mg g^–1^)	3.4	3.3	3.7	4.3	3.3
NCGM (mg g^–1^)	16.1	16.2	16.0	18.1	18.3
N uptake (g)	0.83	0.85	1.00	1.00	0.63
NUtE (g g^–1^)	42.84	41.47	42.97	34.56	37.53
NHI (%)	69	66	67	62	68
GHI	14	14	13	24	16
SDS-Sed	4.8	4.0	4.3	5.0	5.0
***High-N***					
Yield (kg ha^–1^)	7,391	7,320	7,098	5,483	5,567
NUE (kg ha^–1^ grain/kg ha^–1^ N supply)	53.95	53.43	51.81	40.03	40.64
NCPH (mg g^–1^)	15.7	15.6	15.8	16.3	15.6
NCPA (mg g^–1^)	11.8	11.3	12.1	12.1	12.9
NCPM (mg g^–1^)	4.5	3.7	5.8	5.2	4.1
NCGM (mg g^–1^)	18.9	18.4	17.9	19.1	19.6
N uptake (g)	1.56	1.29	1.53	1.30	1.35
NUtE (g g^–1^)	35.11	36.76	33.45	31.24	34.22
NHI (%)	65	68	57	58	66
GHI	20	17	9	17	19
SDS-Sed	4.8	5.5	5.3	4.8	5.3

*Nitrogen concentration at heading (NCPH; mg g^–1^), anthesis (NCPA; mg g^–1^), maturity (NCPM; mg g^–1^), in grains (NCGM; mg g^–1^), nitrogen uptake (g), nitrogen utilization (NUtE; g g^–1^), and nitrogen harvest index (NHI; %) determined from in season tissue analysis for five lines. Grain hardness index (GHI) based on single kernel characterization (SKCS). SDS Sedimentation (SDS-Sed) based on whole grain flour meal.*

Spike traits were investigated by measuring SPL and the KNS in both environments. The effect of N and G were significant on SPL and KNS (Supplementary Table S3). SPL ranged from 5.9 to 10.5 cm (Supplementary Table S2). The mean SPL was 7.8 cm in low N and 8.4 cm in high N. Positive correlation was observed between SPL and KNS at 0.53 in high N and 0.59 in low N, respectively (Supplementary Table S4). The mean KNS in high N was 32, in comparison to the mean KNS of 29 in low N. However, the range was similar under both N levels, from 20 to 50 KNS. PU28 produced the most KNS in high N with average of 41, and PU15 produced the most KNS under low N (Supplementary File S1). The percent reduction of SPL and KNS from high-N to low-N treatments were, on average, 7.7 and 10.3%, respectively. In most cases, larger SPL values were associated with larger KNS values, suggesting that the length of the spike could be a primary determinant of the number of kernels per spike.

Lines were significantly different for fruiting efficiency (FE) (Supplementary Table S3); however, N did not affect FE. FE was highly heritable across environments (*H*^2^ > 0.50) (Supplementary Table S2). In high N, FE showed a mean of 87 kernels per gram of dry matter spike at anthesis (range 21–186) (Supplementary Table S2). Genotypes PU02 and PU20 had the lowest FE of 57 and 62 in high-N environment, well below the average. PU07 and PU19 showed FE above 100 in both low-N and high-N treatment (Supplementary File S1).

### In-Tissue Nitrogen Analysis

N treatment had significant effects on N concentration in phytomass at heading, anthesis, and maturity, as well as in grains for the 5 subset genotypes (Supplementary Table S3). On average, N concentration in biomass at heading was 11.1 mg g^–1^ in low N (Supplementary Table S2) where genotype PU17 showed the maximum in-biomass N concentration (Table 2). In high N, plants were able to accumulate N concentration of 15.8 mg g^–1^ in biomass at heading (Supplementary Table S2). The amount of in-biomass N concentration decreased to 8.8 and 12.1 mg g^–1^ by anthesis in low-N and high-N treatments and in-phytomass N concentration decreased to 3.5 and 4.7 mg g^–1^ by maturity in low-N and high-N treatments, respectively (Supplementary Table S2).

From anthesis to maturity, the amount of N in phytomass decreased. The effect of N and Y was significant for N concentration at anthesis and maturity (Supplementary Table S3) where PU21 displayed the largest loss of 8.8 mg g^–1^ N from anthesis to maturity in high N, while PU15 lost 5.3 mg g^–1^ in low N (Table 2). This signifies the translocation of N into the grains. Genotypes were only significantly different at maturity stage for N concentration in phytomass and in grains (Supplementary Table S3). The maximum NHI of 69% was observed in PU08 in low N. While the minimum NHI of 57% was observed in PU15 in high N (Table 2). The sum of N in phytomass and grain at maturity was approximately 22.0 mg g^–1^, on average (Supplementary Table S2). The total N at anthesis was approximately 10.5 mg g^–1^ across environments. We observed that pre-anthesis N concentration was correlated with grain N concentration (*r* = 0.51; *p*-value < 0.001) among the 5 lines (data not shown).

### Nitrogen Use Efficiency

Nitrogen use efficiency was estimated for all 30 lines across N treatments. N, G, Y × G, N × G, and Y × N × G were significant for NUE (Supplementary Table S3). Due to the level of N application, and method of calculation, NUE estimates were higher in low N (Supplementary Table S2). For example, NUE averaged 209.92 kg ha^–1^ grain per kg ha^–1^ N supplied in low-N environment. PU03 had the lowest NUE of 179.78 kg ha^–1^ grain per kg ha^–1^ N, with PU13 the highest at 243.62 kg ha^–1^ grain per kg ha^–1^ N (Supplementary File S1). In high N, NUE averaged 46.05 kg ha^–1^ N. PU08, PU10, and PU15 had the greatest NUE in high N (Table 2 and Supplementary File S1). We further quantified N uptake, NUtE, and NHI in 5 selected genotypes in this study (Table 2). The effect of N was significant on N uptake (Supplementary Table S3). N uptake average 1.42 and 0.87 g in high N and low N, respectively (Supplementary Table S2). This was a 38% reduction in whole plant N uptake. However, the effect of G and G × N was not significant, indicating that lines responded similarly to their N uptake across the two environments (Supplementary Table S3). The effects of Y, N, G, and Y × G were significant on NUtE (Supplementary Table S3). NUtE was significantly greater in low N (compared to high N) by 14% (Supplementary Table S2). The effects of N, G, Y × G, and N × G was significant on NHI (Supplementary Table S3). NHI ranged from 42 to 75% across years and environments.

### Glutenin Subunits and the Rye Translocation

Loci for HMW glutenin subunits *Glu-A1*, *Glu-B1*, and *Glu-D1* and LMW subunits *Glu-A3, Glu-B3*, and *Glu-D3* and presence of 1B/1R translocation (Table 3) were characterized (Supplementary Figure S1). In the thirty lines tested, the common *Glu-A1* allele was the *1* subunit with only six genotypes possessing the *2*^∗^ allele. The variants observed in *Glu-B1* locus were *7, 7* + *8*, *7* + *9*, *13* + *16*, and *32* + *33* subunits. Two alleles *2* + *12* and *5* + *10* were found for *Glu-D1* locus at almost equal frequency. For LMW, the *Glu-A3c* subunit and *Glu-D3a* subunit were the most frequent (Table 3), while *Glu-B3* showed a wide allelic variation. The 1B/1R rye translocation was identified in 17 out of 30 genotypes. When we compared genotypes with translocation with those without the translocation by using two-sample *t*-test, the difference was not significant (*p*-value > 0.05) (data not shown). Genotypes with the 1B/1R translocation varied in allelic variation for HMW and LMW subunits (Table 3).

**TABLE 3:** Allelic variation of high (HMW) and low molecular weights (LMW) for glutenin subunits and presence of 1B/1R translocation for each line.

	Low-N	High-N	HMW			LMW				Low-N		High-N	
Germplasm	Yield	Yield	Glu-A1	Glu-B1	Glu-D1	Glu-A3	Glu-B3	Glu-D3	Translocation	GHI	SDS-Sed	GHI	SDS-Sed
PU01	5,001	6,296	1	7	2 + 12	f	j	a	1B/1R	10	4.0	13	4.8
PU02	5,405	6,292	2*	32 + 33^†^	5 + 10	c	j	b	1B/1R	6	4.8	12	5.5
PU03	4,494	5,842	1	7	5 + 10	c	f,g	a	–	9	4.8	12	5.8
PU04	5,340	5,900	1	7	5 + 10	d^†^	b	a	–	9	4.0	13	4.3
PU05	5,426	6,780	1	7 + 9	2 + 12	d	f,g,j^†^	c/b	1B/1R	2	6.0	4	6.3
PU06	5,571	6,485	1	13 + 16^†^	2 + 12	c	f,g	a	–	7	4.3	13	5.8
PU07	4,668	6,328	1	7	2 + 12	f	j	a	1B/1R	13	4.3	16	5.0
PU08	5,699	7,392	2*	7	2 + 12	g	j	a	1B/1R	14	4.8	20	4.8
PU09	5,875	6,479	1	7	2 + 12	c	b	a	1B/1R	10	5.0	12	6.3
PU10	5,528	7,320	2*	7 + 9	2 + 12	g	j	a	1B/1R	14	4.0	17	5.5
PU11	5,269	6,105	1′^±^	13 + 16	5 + 10	c	h	a	–	20	6.3	17	7.3
PU12	5,270	5,656	1	7	5 + 10^†^	c	f^†^	b^†^	1B/1R	12	5.3	17	6.3
PU13	6,090	6,817	1′^±^	13 + 16	5 + 10	c	h	a	–	16	5.5	17	7.0
PU14	4,917	6,151	0	7 + 8	2 + 10.1 ^±^	c	g	a	–	19	5.5	20	7.0
PU15	5,752	7,099	1	7	2 + 12	c	b	a	1B/1R	13	4.3	9	5.3
PU16	5,638	6,710	1	7 + 9^†^	2 + 12^†^	c	j^†^	c^†^	1B/1R	13	3.8	16	4.0
PU17	4,696	5,484	1	7	2 + 12	c	j	a	1B/1R	24	5.0	17	4.8
PU18	5,870	6,707	1	7 + 8	2 + 12/5 + 10	c	b	b	–	12	4.5	9	5.0
PU19	5,650	6,148	2*	7 + 9	2 + 12	c	j	c	1B/1R	23	4.8	29	6.3
PU20	5,742	6,676	2*^†^	7 + 9	2 + 12^†^	c	h^†^	a	–	11	3.8	16	5.0
PU21	4,928	5,568	1	7^†^	2 + 12	f	j^†^	a	1B/1R	16	5.0	19	5.3
PU22	5,617	6,242	1	7 + 8	2 + 12	c	b′	a	–	22	4.8	18	5.8
PU23	5,619	6,402	1	7	2 + 12	d	b′	a	–	12	5.0	14	5.3
PU24	4,719	5,851	1′^±^	13 + 16^†^	2 + 12	c	h/b	a	–	25	5.3	31	4.8
PU25	5,979	6,866	2*	7	2 + 12	g	j	a	1B/1R	17	4.3	21	5.3
PU26	5,802	6,170	1	7 + 9^†^	2 + 12^†^	c	j	c^†^	1B/1R	19	3.8	25	4.3
PU27	5,358	6,230	1	7 + 8	5 + 10^†^	c	b	b^±†^	–	15	5.0	16	5.3
PU28	4,901	5,938	1	7 + 8/32 + 33	5 + 10/2 + 12	c	f, g, j^†^	b^†^	1B/1R	6	4.8	9	4.8
PU29	5,040	6,059	1	7 + 9	2 + 12	c	j	c^†^	1B/1R	16	5.3	19	4.8
PU30	5,080	6,065	1	7 + 8	5 + 10/2 + 12	d	b′	b	–	7	4.5	12	5.0

^±^Indicates similar to the allele showed but not confirmed with a proper check. ^†^Indicates that the allele was not identified with certainty. Grain hardness index (GHI) and SDS-Sedimentation (SDS-Sed) evaluated under both nitrogen environments for each line. *Indicates of an allele variation.

### Grain Quality Indicators

The GHI values greater than 59 are indicative of hard while GHI values less than 33 specify soft endosperms. Because we analyzed only single replicate grains with SKCS, we could not perform ANOVA or any significance test among genotypes. GHI averaged 13.8 ± 1.03 (standard error of the mean) in low N. In high N, GHI averaged 16.1 ± 1.05 (Table 3). PU24 showed maximum GHI values of 25 and 31 in low N and high N, respectively. In contrast, PU05 showed the minimum GHI values less than five in both treatments.

For SDS-sedimentation, higher values indicate better bread-making quality (Moonen et al., 1982). SDS tested whole meal flour samples of each line performed in duplicate showed sedimentation mean of 5.4 ± 0.15 in high N in contrast to 4.7 ± 0.12 sedimentation mean observed in low N (Table 3). PU16 showed minimum SDS-sedimentation while PU11 showed the maximum.

Germplasm with the 1B/1R translocation showed a lower grain hardness and lower SDS-sedimentation (Table 3). For example, PU05 and PU16 had the minimum GHI and the minimum SDS-sedimentation across environments, respectively, while PU11 and PU24 which do not carry the translocation show maximum GHI and SDS-sedimentation for whole grain flour meal. PU10 and PU15 exhibit the translocation and were among the highest yielding lines in high N and low N, with lower protein in both environments and a lower SDS-sedimentation score than average in low N (Table 3).

### Nitrogen × Genotype Interaction

**FIGURE 1:**
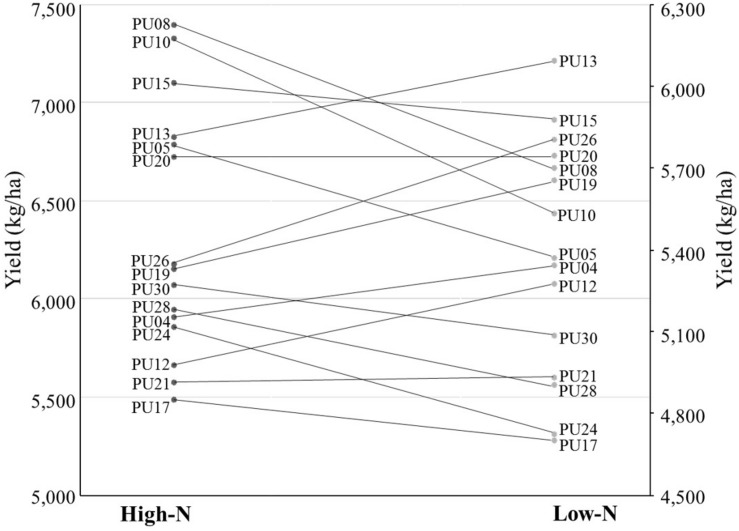
Genotype ranking and interactions based on grain yield (kg ha^– 1^) in low-N and high-N environment for 15 out of 30 genotypes.

Five traits including grain yield, grain number, number of spikes, nitrogen use efficiency, and nitrogen harvest index showed significant N × G interaction effect (Supplementary Table S3), indicating that lines performed differently in response to nitrogen environments. In particular, when we assessed grain yield with ranks, a cross over interaction was observed for lines PU08 and PU13. PU08 was the first rank line in the high-N environment while PU13 was the first rank in the low-N environment (Figure 1). The change was evident as only 4 of 30 genotype held the same rank across environments. One specific genotype, PU26, is an example of the importance of phenotyping in low input environments. Under high N, PU26 yielded 6,170 kg ha^–1^, below average, and ranked as the 18th best genotype based on yield performance. However, in low N, PU26 yielded 5,802 kg ha^–1^, above average, and moved up 12 spots to the 6th best yielding genotype (Supplementary File S1). The change in ranking was indicative of genotype by nitrogen interaction.

### Principal Component Analysis (PCA) – Biplot Analysis

**FIGURE 2:**
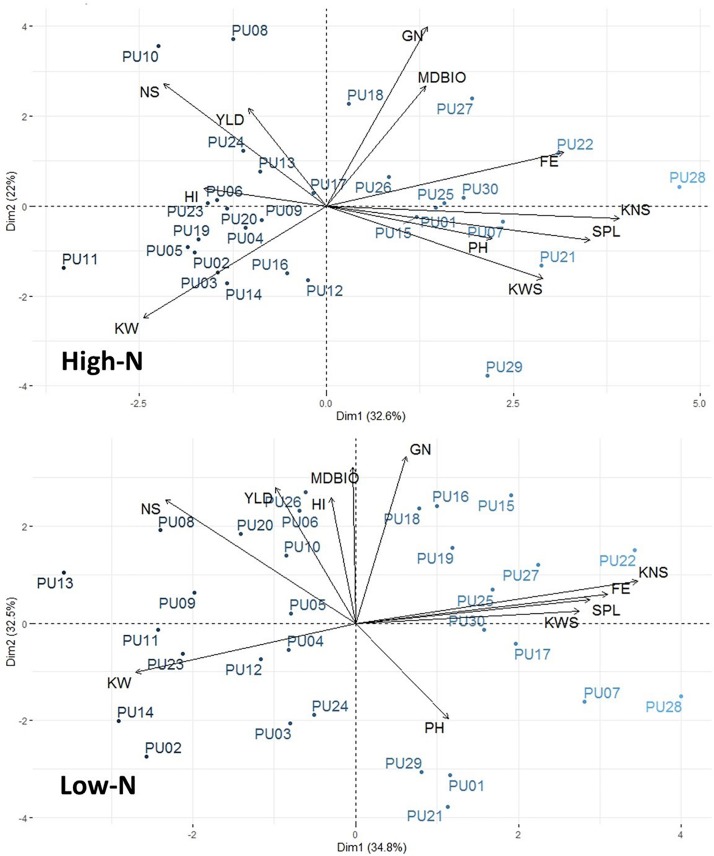
Principal component analysis (PCA)-biplot analysis among 12 agronomic traits and 30 genotypes. PCA-biplots were performed in both high-N and low-N environments.

The interrelationship among traits and genotypes in the form of biplots in each environment is shown in Figure 2. Principal component analysis (PCA) was performed on the 12 traits measured and all 30 lines in both environments. In low N, PC1, and PC2 explained 34.8 and 32.5% of phenotypic variations, respectively. In high N, PC1 and PC2 explained 32.6 and 22.0% of phenotypic variation, respectively. The number of spikes was significantly and positively associated with grain yield in both environments (Figure 2 and Supplementary Table S4). Kernel weight was not positively associated with any other trait but had significant negative correlations with harvest index and fruiting efficiency. Lines are also visually shown in PCA-biplot. Two high yielding lines in both environments, PU08 and PU10, were in the same direction as grain yield and number of spikes.

## Discussion

Of the estimated 31.8 million acres of winter wheat planted in 2019, approximately 5.54 (∼17%) million acres are estimated to be planted as soft-red winter wheat in the eastern United States. A record low harvest area is expected in New Jersey, Ohio, and Virginia (USDA, 2019). The decline in wheat cultivation area in the United States is due to an increase in acreage and production of maize and soybean. In maize, nitrogen dynamics and optimizations under varying environments have been studied extensively to increase productivity with efficient fertilization, management, and less environmental footprint (Bänziger et al., 1997; Ciampitti and Vyn, 2012). Studies in wheat took a variety of objectives from improving wheat for low-nitrogen input in order to reduce environmental impacts (Ortiz-Monasterio et al., 1997; Delogu et al., 1998; Le Gouis et al., 2000; Brancourt-Hulmel et al., 2005), breeding for productivity gains and cost-effectiveness under low input environments (Bänziger and Cooper, 2001), and nitrogen use efficiency in soft-red winter wheat (Van Sanford and MacKown, 1986; Hitz et al., 2017; Brasier et al., 2018). The ability to identify nitrogen efficient soft-red winter wheat germplasm will have the potential to reduce N applications, therefore saving time, resources, and management costs.

### Yield and Yield Component Responses

The rank change of lines across environments, e.g., from high N to low N (Figure 1), can indicate the potential profit loss or gain. For example, the profit made by PU17, which yielded 4,696 and 5,484 kg ha^–1^ under low N and high N, would be below the average profit margins across all 30 lines and displays the potential loss in comparison to other higher yielding lines (Supplementary File S1). This data seems to suggest breeding specifically for separate environments by using beneficial founder individuals for each environment. A PCA-biplot that shows trait and line associations (Figure 2), can be useful for shortlisting of founder individuals. For example, in low N, unlike in high N, the biomass at maturity has a close association and higher correlation (Supplementary Table S4) with grain yield, showing that, under limited nitrogen, the decreases of biomass (tillers and leaves), is the bottleneck for grain production later in the season. Therefore, it seems that the negative effect of low N is through reduction in canopy size and radiation use. Yield potential is expressed as a function of light interception, radiation use efficiency, and harvest index, where the critical underlying trait common to all three components is above-ground plant biomass. An increase in biomass is associated with an increase in radiation use efficiency, grain number, and ultimately grain yield (Reynolds et al., 2005). In spring wheat, Caviglia and Sadras (2001) observed nitrogen deficiency reduced light interception and radiation use efficiency, ultimately because of smaller leaf area index due to decrease tillering and less shoot dry matter (biomass). Calderini et al. (1997) identified wheat cultivars reached a maximum leaf area index between the booting and terminal spikelet growth stage, implying the importance of establishing a wheat canopy earlier in the growth season as leaf area index and dry matter decreases post-anthesis when the wheat transitions from vegetative growth to reproductive growth for grains.

In our study, the difference in spike number can be attributed to the lack of tiller initiation in the spring or the loss of an emerging tiller in winter. The decreases in biomass due to low-N treatment resulted in reduction of grain number via decreases of number of spikes, and kernel per spike, similar to previously reported observations (Le Gouis et al., 2000; Terrile et al., 2017). Grain number, as an important yield component, is positively related to pre-anthesis dry matter accumulation (Duan et al., 2018) and was shown to respond directly to N supply to the spike (Abbate et al., 1995). Our results indicate grain number and biomass are highly correlated (Supplementary Table S4) and are associated with genotypes producing more grain in low and high N (Figure 2). Despite responsiveness of grain number, our study indicated that kernel weight is more stable under environmental conditions with higher heritability (*H*^2^ = 0.88 and 0.89), implying that the physiological mechanisms that control grain filling are able to fill the number of grains that were determined earlier. Even though a contradicting report of kernel weight was described as the main determinant of grain yield (Major et al., 1988), we observed grain number as the primary contributor for grain yield. Similar to our observation, other physiological studies reported similar behavior for environmental responsiveness of grain number and kernel weight (Sadras and Slafer, 2012; Slafer et al., 2014; Ferrante et al., 2017).

### End-Use Quality Determinants

One aspect of genotypic differences in responses to low N is end-use quality traits. Protein content, gluten quality, and endosperm texture in wheat are the driver of end-use products. Several studies evaluated the relationship between grain yield to protein content and quality. For example, experimental evidence is indicative of a negative correlation between grain yield and protein (Cooper et al., 2001; Magallanes-López et al., 2017). We used several measures to understand the dynamics of protein quality under the two contrasting N regimes.

Contrary to changes that we observed for grain yield under different N management, our study only indicated a slight decrease in SDS-sedimentation and grain hardness index. This is an opportunity for developing low-N efficient soft-red winter wheat breeding because these traits were minimally affected by the lack of sufficient N. Contrary to our results of soft-red winter wheat, N fertilizer was previously shown to have significant effect on SDS sedimentation in hard wheat (Luo et al., 2000; Saint Pierre et al., 2008).

Gluten quality is a function of allelic variation of HMW and LMW subunits. For example, *Glu-A1(2^∗^)* and *Glu-D1(5* + *10)* HMW subunits are considered high gluten quality alleles. Line PU02 revealed high yield and possessed *Glu-A1(2^∗^)* and *Glu-D1(5* + *10)* HMW subunits. One of the highest yielding lines under low N, PU15, possessed *Glu-A1(1)* and *Glu-D1(2* + *12)* subunits, which are not considered the highest glutenin quality alleles. Selection of lines as breeding parents with reasonable yield under low N condition and high glutenin subunits as parents of breeding populations, may be a way to maintain the quality under low N in the breeding population.

Germplasm with the 1B/1R translocation showed a lower grain hardness and lower SDS-sedimentation. For example, PU05 and PU16 had the minimum GHI and the minimum SDS-sedimentation across environments, respectively, while PU11 and PU24 which do not carry the translocation show maximum GHI and SDS-sedimentation for whole grain flour meal. PU10 and PU15 exhibit the translocation and were among the highest yielding lines in high N and low N (Figure 1), with lower protein in both environments and a lower SDS-sedimentation score than average in low N (Table 3). Morris and Paulsen (1985) analyzed hard winter wheat under two contrasting treatments. In deficient N, the low levels of vegetative N resulted in a significant decreased in total grain N after anthesis. In comparison, high N maintained 37 mg N plant^–1^ throughout grain filling but increased grain N dramatically (Morris and Paulsen, 1985). Parts of the N that is in the grain comes from senescence of leaves (remobilization of existing N compounds) (Hawkesford, 2014). Tolley and Mohammadi (2020), showed significant differences for grain N at maturity in seven diverse wheat accessions. The grain N in low-N treatment was 23.3 mg g^–1^ while grain N in high-N environment was 27.8 mg g^–1^. Our study did not detect any significant genotypic variation of N uptake in spite of previous studies showing genetic variation in nitrogen uptake and assimilation previously described in wheat (Cox et al., 1985; Ortiz-Monasterio et al., 1997; Le Gouis et al., 2000).

### Breeding for Low-N Environments

A comparative view of the crop produced per nitrogen used in this study indicates that breeding and selection for performance under low-N environment has the potential for minimizing N use and environmental impacts. In our study each additional kg ha^–1^ of spring N fertilizer resulted in a grain yield increase of 9 kg ha^–1^, with the G × N effect for grain yield being significant, indicating that lines responded differently (Table 1). For example, PU10 responded maximally and PU04 responded minimally by increasing 16 and 5 kg ha^–1^ of yield per each kg ha^–1^ of nitrogen applied (Supplementary File S1).

Most breeding programs and variety testing are historically performed under optimal conditions and sufficient N applications for evaluating yield potential. N applications have the negative environmental impact of leaching, pollution, and runoff into the water, as nitrate is the most commonly detected agricultural chemical in the water. Wu et al. (1996) estimated an average annual runoff and leaching of 4.47 and 4.57 kg N ha^–1^, respectively, in the midwestern and northern plain regions under corn, sorghum, soybean, wheat, or legume hay cultivation, accounting for about 5.5 and 5.6% of N applied.

This result indicates that establishing breeding and selection for specifically performance under low-N cropping systems has the potential to produce reasonably well under low-N conditions while decreasing the environmental footprint. The former was evident by changes in rank analysis of lines in both environments (Figure 1). Change of rank in differential environments was previously used in drought (Li et al., 2011; Lopes et al., 2014), salinity (Salam et al., 1999; Chamekh et al., 2015), and other nutrient deficiencies (Torun et al., 2000; Murphy et al., 2008; Zhao et al., 2018), to postulate a need for environment specific management and breeding practices. For example, van Bueren and Struik (2017) described breeding for grain crops and vegetables under diverse N management for genotype adaptation and interaction with availability of N.

Our data seems to suggest that the lines PU05, PU08, PU10, PU13, PU15, PU19, PU14, PU20, and PU26 have the potential to be the founder of a breeding population for low-N environment (Figure 1). For this selection we used criteria such as higher ranks in low-N conditions, higher kernel per spike in low-N, higher kernel weight, superior *Glue-A1* (*2*^∗^) allele, the rye 1B/1R translocation, and higher NHI and FE. Another related trait that can help wheat breeding for low-N system is the use higher grain protein content trait. It has been shown that greater translocation of nitrogen to grains from increased fertilizer N results in a higher grain protein concentration (Delogu et al., 1998; Saint Pierre et al., 2008). A grain protein content (GPC) locus, *GPC-B1*, has been identified on chromosome 6B in wheat (Distelfeld et al., 2006). *Gpc-B1* increases protein content via N remobilization from leaves and senescence (Uauy et al., 2006).

## Conclusion

In conclusion, we propose the first ideotype for breeding N-efficient cultivars specifically for the United States midwest wheat. In soft-red winter wheat, where grain yield and relatively lower grain protein content is desired, we believe that in-tissue concentration of nitrogen, which traditionally represents uptake and utilization of N, may not be a good indicator of nitrogen use efficiency.

In fact, a superior and N-efficient genotype is one which uses the available N to produce a canopy allowing for maximum radiation use efficiency, producing dry matter that is required for fertile tiller and grain numbers. Therefore, for a grain crop where protein content is not critical, a good indicator of nitrogen use efficiency is fixation of carbon, efficient use of radiation, and developing a productive canopy, per unit of nitrogen used. The rank differences among lines in contrasting environments is a testament to the opportunity to select and breed for more crop per same N (or same crop with less or optimized N). In this context, the success of wheat breeding for N-deficient environments needs management strategies that enable supplying continuous availability of N in the field post-anthesis and during grain fill.

Our study resulted in identification of traits and variants that will lead to increases of yield and maintaining of yield under lower nitrogen conditions, and therefore can be regarded as “the breeder’s toolkit for developing N-efficient soft-red winter wheat varieties.” For breeding soft-red winter wheat for high-N environment, PU08, PU10, and PU15 would be advantageous due to responsiveness to N with significant increases in grain number, biomass, and number of spikes, which led to the increase in grain yield. Since N treatment did not significantly impact end-use quality of the grains, N management in soft-red winter wheat can focus on the best practices for canopy enhancement, grain number per unit area, and yield.

## Germplasm and Detailed Data

Seed and detailed data for each line evaluated in this study is available via the wheat breeding program, Purdue University.

## Data Availability Statement

All datasets generated for this study are included in the article/Supplementary Material.

## Author Contributions

BR and MM planned and designed the research, and wrote the manuscript. BR collected field measurements and analyzed the data. CG performed gluten quality measurements. BR, CG, and MM edited and revised the manuscript. All authors read and approved the manuscript.

## Conflict of Interest

The authors declare that the research was conducted in the absence of any commercial or financial relationships that could be construed as a potential conflict of interest.
