# Supplementary material for: Cultivar, Trait and Management System Selection to Improve Soft-Red Winter Wheat Productivity in the Eastern United States
Source: Front Plant Sci. 2020 Mar 31;11:335. doi: 10.3389/fpls.2020.00335 (PMC7136473; doi:10.3389/fpls.2020.00335)

**Supplementary Table S1:** Average temperature and precipitation for the duration of the study. Historical averages based on previous 30 years from the National Weather Service.

|  | **Temperature (°C)** | | |  | **Precipitation (mm)** | | |
| --- | --- | --- | --- | --- | --- | --- | --- |
| **Month** | **2016- 2017** | **2017-2018** | **Historical** |  | **2016- 2017** | **2017-2018** | **Historical** |
| September | 20.8 | 19.3 | 19.4 |  | 81.0 | 50.5 | 79.5 |
| October | 15.1 | 14.5 | 12.8 |  | 32.8 | 68.1 | 79.2 |
| November | 8.3 | 5.3 | 6.5 |  | 135.9 | 125.8 | 94.0 |
| December | -1.9 | -1.4 | -0.2 |  | 58.9 | 20.6 | 80.5 |
| January | -0.2 | -4.7 | -2.2 |  | 111.7 | 39.9 | 67.6 |
| February | 4.7 | 0.4 | 0.1 |  | 19.9 | 139.8 | 58.9 |
| March | 5.6 | 2.8 | 5.7 |  | 109.1 | 79.1 | 90.4 |
| April | 13.5 | 6.8 | 11.7 |  | 108.7 | 73.7 | 96.8 |
| May | 15.7 | 21.1 | 17.1 |  | 175.3 | 93.7 | 128.3 |
| June | 22.2 | 22.8 | 22.2 |  | 135.4 | 157.8 | 107.9 |

**Supplemental Table S2**: Mean, standard deviation (sd), and range of 14 agronomic traits and 7 in tissue nitrogen analysis traits in both environments. Heritability (H^2^) calculated for all 30 genotypes per nitrogen treatment.

|  | **High-N** | | | **Low-N** | | |
| --- | --- | --- | --- | --- | --- | --- |
| **Trait – All 30 Genotypes** | **Mean ± sd** | **Range** | **H^2^** | **Mean ± sd** | **Range** | **H^2^** |
| *Plant Development* |  |  |  |  |  |  |
| Days to Heading (HD) | 130 ± 5.53 | 119 - 137 | 0.66 | 130 ± 5.57 | 119 - 137 | 0.70 |
| Days to Maturity (MD) | 168 ± 3.53 | 162 - 175 | 0.55 | 167 ± 3.4 | 162 - 174 | 0.71 |
| Biomass at Maturity (g) (MD_BIO_) | 109.02 ± 28.57 | 46.86 - 193.59 | 0.19 | 87.51 ± 28.07 | 21.09 - 157.49 | 0.20 |
| Plant height (cm) (PLH) | 89.11 ± 9.43 | 67.25 - 112.75 | 0.77 | 81.42 ± 9.95 | 54.50 - 104.75 | 0.62 |
| *Yield Components* |  |  |  |  |  |  |
| Yield (kg ha^-1^) (YLD) | 6,335 ± 824.04 | 3,799 – 8,090 | 0.46 | 5,359 ± 888.4 | 2,965 – 7,640 | 0.41 |
| NUE (kg ha^-1^ grain / kg ha^-1^ N supply) | 46.05 ± 6.70 | 27.73 – 59.05 | 0.46 | 209.92 ± 46.67 | 118.62 – 305.61 | 0.41 |
| Grain Number per area (GN) | 1,312 ± 317.99 | 538 – 2,128 | 0.23 | 1,037 ± 340.94 | 297 – 1,842 | 0.27 |
| Number of Spikes per area (NS) | 58 ± 11.24 | 32 - 100 | 0.48 | 50 ± 11.03 | 25 - 94 | 0.55 |
| Kernel Weight (mg) (KW) | 36 ± 4.4 | 25 - 46 | 0.88 | 36 ± 3.9 | 28 - 47 | 0.89 |
| Spike length (cm) (SPL) | 8.4 ± 0.8 | 6.3 - 10.5 | 0.75 | 7.8 ± 0.7 | 5.9 - 10.0 | 0.63 |
| Kernel number per spike (KNS) | 32 ± 6.08 | 15 - 49 | 0.52 | 29 ± 6.18 | 14 - 48 | 0.52 |
| Kernel weight per spike (g) (KWS) | 1.02 ± 0.21 | 0.51 - 1.66 | 0.51 | 0.91 ± 0.17 | 0.47 - 1.34 | 0.38 |
| Fruiting efficiency (grains g^-1^) (FE) | 87 ± 37.93 | 21 - 186 | 0.57 | 85 ± 39.56 | 23 - 210 | 0.56 |
| Harvest index (HI) | 0.44 ± 0.05 | 0.27 - 0.55 | 0.22 | 0.38 ± 0.07 | 0.21 - 0.55 | 0.15 |
| **Trait – 5 Subset Genotypes** |  |  |  |  |  |  |
| *Nitrogen Analysis* |  |  |  |  |  |  |
| Nitrogen concentration of Phytomass at Heading (NCPH) (mg g^-1^) | 15.8 ± 1.9 | 11.0 - 20.8 | - | 11.1 ± 2.3 | 7.9 - 17.9 | - |
| Nitrogen concentration of Phytomass at Anthesis (NCPA) (mg g^-1^) | 12.1 ± 2.7 | 8.1 – 17.8 | - | 8.8 ± 1.8 | 6.3 – 16.6 | - |
| Nitrogen Concentration of Phytomass at Maturity (NCPM) (mg g^-1^) | 4.7 ± 1.6 | 2.6 – 10.7 | - | 3.5 ± 0.8 | 2.4 – 6.4 | - |
| Nitrogen concentration of Grains at Maturity (NCGM) (mg g^-1^) | 18.7 ± 2.6 | 13.5 – 23.4 | - | 16.9 ± 2.1 | 12.8 – 20.3 | - |
| N uptake (g g^-1^) | 1.42 ± 0.34 | 0.69 - 2.62 | - | 0.87 ± 0.29 | 0.42 - 1.53 | - |
| NUtE (g g^-1^) | 34.13 ± 5.99 | 18.10 - 45.72 | - | 39.78 ± 6.14 | 24.76 - 51.58 | - |
| NHI (%) | 63 ± 7 | 42 - 72 | - | 66 ± 6 | 46 - 75 | - |

**Supplementary Table S3**: ANOVA for year (Y), nitrogen level (N), genotype (G), and interactions for measured traits. ANOVA performed on all 30 lines except for last 7 traits relating to N analysis. Significance: < 0.001 = ***, <0.01 = **, < = 0.05*, and > 0.05 = ns.

| **Trait – All 30 genotypes** | **Y** | **N** | **Y x N** | **G** | **Y x G** | **N x G** | **Y x N x G** |
| --- | --- | --- | --- | --- | --- | --- | --- |
| Days to Heading (HD) | *** | ns | ns | *** | *** | ns | ns |
| Days to Maturity (MD) | *** | *** | ns | *** | ** | ns | ns |
| Biomass at Maturity (g) (MD_BIO_) | ** | * | ns | * | ns | ns | * |
| Yield (kg ha^-1^) (YLD) | ns | * | ns | *** | *** | * | ns |
| NUE (kg ha^-1^ grain / kg ha^-1^ N supply) | ns | *** | ns | *** | *** | *** | * |
| Spike length (cm) (SPL) | ** | *** | ns | *** | * | ns | ns |
| Kernel number per spike (KNS) | *** | *** | ns | *** | ns | ns | ns |
| Kernel weight per spike (g) (KWS) | *** | *** | * | *** | ** | ns | ns |
| Grain Number per area (GN) | ns | ** | ns | *** | ns | * | * |
| Number of Spikes per area (NS) | ns | ns | ns | *** | ns | * | * |
| Kernel Weight (mg) (KW) | *** | ns | ns | *** | *** | ns | ns |
| Fruiting efficiency (grains g^-1^) (FE) | *** | ns | ns | *** | *** | ns | ns |
| Harvest index (HI) | *** | ns | ns | *** | * | ns | ns |
| Plant height (cm) (PLH) | *** | ** | ns | *** | ns | ns | ns |
| **Trait – 5 subset genotypes** |  |  |  |  |  |  |  |
| Nitrogen Concentration of Phytomass at Heading (NCPH) (mg g^-1^) | * | *** | ns | ns | ns | ns | ns |
| Nitrogen Concentration of Phytomass at Anthesis (NCPA) (mg g^-1^) | *** | *** | ns | ns | ns | ns | ns |
| Nitrogen Concentration of Phytomass at Maturity (NCPM) (mg g^-1^) | ** | ** | ns | ** | * | ns | ns |
| Nitrogen Concentration of Grains at Maturity (NCGM) (mg g^-1^) | *** | *** | * | *** | ** | ns | ns |
| N uptake (g) | ns | ** | ns | ns | ns | ns | * |
| NUtE (g g^-1^) | *** | *** | ns | *** | *** | ns | ns |
| NHI (%) | ns | * | ns | *** | ** | * | ns |

**Supplementary Table S4**: Correlation table of Pearson correlation coefficients and significant p-values of correlations. Significance: < 0.001 = ***, <0.01 = **, < = 0.05. Upper right triangle represents low-N and lower left triangle represents high-N environment. Ordered by first principal components. Abbreviations as follows: KW = kernel weight, PLH = plant height, HI = harvest index, KWS = kernel weight per spike, NS = number of spikes per unit area, MDBIO = biomass at maturity, SPL = spike length, FE = fruiting efficiency, NUE = nitrogen use efficiency, YLD = yield, GN = grain number per unit area, and KNS = kernel number per spike.

|  | KW | PLH | HI | KWS | NS | MDBIO | SPL | FE | NUE | YLD | GN | KNS |
| --- | --- | --- | --- | --- | --- | --- | --- | --- | --- | --- | --- | --- |
| KW |  | 0.45* | -0.45*** | 0.09 | -0.09 | 0.25* | -0.26 | -0.75* | -0.16 | -0.16 | -0.34 | -0.54 |
| PLH | 0.55 |  | -0.57 | 0.19 | -0.06 | 0.43 | 0.11 | -0.52* | 0.10 | 0.10 | -0.03 | -0.19 |
| HI | -0.32*** | -0.52 |  | 0.00 | 0.04 | -0.33 | 0.13 | 0.51** | 0.05 | 0.05 | 0.31 | 0.28 |
| KWS | 0.27 | 0.32 | -0.09 |  | -0.11 | 0.3 | 0.46 | 0.66 | 0.10 | 0.05 | 0.23 | 0.68 |
| NS | -0.25 | -0.22 | 0.03 | -0.31 |  | 0.51 | -0.02 | 0.02 | 0.64* | 0.64* | 0.60 | -0.04 |
| MDBIO | 0.35 | 0.53 | -0.53 | 0.14 | 0.21 |  | 0.18 | -0.32 | 0.44 | 0.44 | 0.71 | 0.04 |
| SPL | -0.18 | -0.12 | 0.22 | 0.37 | -0.09 | 0.01 |  | 0.31 | 0.09 | 0.09 | 0.32 | 0.59 |
| FE | -0.80* | -0.54 | 0.34** | -0.07 | 0.18 | -0.39 | 0.22 |  | 0.10 | 0.10 | 0.23 | 0.66 |
| NUE | -0.11 | -0.12 | 0.22 | -0.05 | 0.36* | 0.10 | 0.02 | 0.16 |  | 1.00 | 0.56 | 0.10 |
| YLD | -0.11 | -0.12 | 0.22 | -0.05 | 0.36* | 0.10 | 0.02 | 0.16 | 1.00 |  | 0.56 | 0.10 |
| GN | -0.30 | 0.01 | 0.06 | -0.09 | 0.42 | 0.66 | 0.12 | 0.21 | 0.34 | 0.34 |  | 0.40 |
| KNS | -0.43 | -0.13 | 0.14 | 0.63 | -0.09 | -0.15 | 0.53 | -0.07 | -0.05 | 0.06 | 0.14 |  |

**Supplemental Figure S1**: Separation of glutenin subunits with SDS-PAGE. Varieties include Purdue Germplasm PU1-3, PU7-9, and OPA (Opata) and PIT (Pitic) were checks for reference. HMW glutenin subunits: OPA (*Glu-A1 2**; *Glu-B1 13+16*; *Glu-D1 2+12*); PU1 (*Glu-A1 1*; *Glu-B1 7*; *Glu-D1 2+12*); PU2 (*Glu-A1 2**; *Glu-B1 32+33*; *Glu-D1 5+10*); PU3 (*Glu-A1 1;* *Glu-B1 7*; *Glu-D1 5+10*); PU7 (*Glu-A1 1*; *Glu-B1 7*; *Glu-D1 2+12*); PU8 (*Glu-A1 2**; *Glu-B1 7*; *Glu-D1 2+12*); PIT (*Glu-A1 1;* *Glu-B1 7+8*; *Glu-D1 2+12*); PU9 (*Glu-A1 1*; *Glu-B1 7*; *Glu-D1 2+12*); PU10 (*Glu-A1 2**; *Glu-B1 7+9*; *Glu-D1 2+12*). LMW glutenin subunits: OPA (*Glu-A3 b*; *Glu-B3 i*; *Glu-D3 a*); PU1(*Glu-A3 f*; *Glu-B3 j*; *Glu-D3 a*); PU2 (*Glu-A3 c*; *Glu-B3 j*; *Glu-D3 b*); PU3 (*Glu-A3 c*; *Glu-B3 f,g*; *Glu-D3 a*); PU7 (*Glu-A3 f*; *Glu-B3 j*; *Glu-D3 a*); PU8 (*Glu-A3 g*; *Glu-B3 j*; *Glu-D3 a*); PU9 (*Glu-A3 c*; *Glu-B3 b*; *Glu-D3 a*); PU10 (*Glu-A3 g*; *Glu-B3 j*; *Glu-D3 a*).


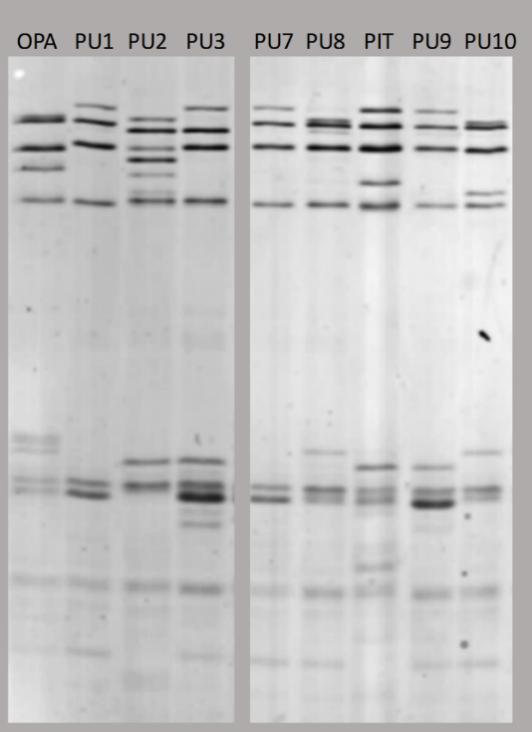

Supplement: Supplementary file 2 [file Data_Sheet_2.docx]
